# Supplementary material for: High Global Health Security Index is a determinant of early detection and response to monkeypox: A cross-sectional study
Source: PLoS One. 2024 Jul 26;19(7):e0307503. doi: 10.1371/journal.pone.0307503 (PMC11280228; doi:10.1371/journal.pone.0307503)
Supplement: S2 Table — (DOCX) [file pone.0307503.s002.docx]

**Table S2.** GHS Index category indicator variables selected for the study

| **Overall score** |  |
| --- | --- |
| **Category** | **Indicator** |
| Prevention | Prevention of the emergence or release of pathogens   - Antimicrobial resistance (AMR) - Zoonotic disease - Biosecurity - Biosafety - Dual-use research and culture of responsible science - Immunization |
| Detection and reporting | Early detection and reporting for epidemics of potential int'l concern   - Laboratory systems strength and quality - Laboratory supply chains - Real-time surveillance and reporting - Surveillance data accessibility and transparency - Case-based investigation - Epidemiology workforce |
| Respond rapid | Rapid response to and mitigation of the spread of an epidemic   - Emergency preparedness and response planning - Exercising response plans - Emergency response operation - Linking public health and security authorities - Risk communication - Access to communications infrastructure - Trade and travel restrictions |
| Health systems | Sufficient and robust health sector to treat the sick and protect health workers   - Health capacity in clinics, hospitals and community care centers - Supply chain for health system and healthcare workers - Medical countermeasures and personnel deployment - Healthcare access - Communications with healthcare workers during a public health emergency - Infection control practices - Capacity to test and approve new medical countermeasures |
| Norms | Commitments to improving national capacity, financing and adherence to norms   - IHR reporting compliance and disaster risk reduction - Cross-border agreements on public health and animal health emergency response - International commitments - Joint External Evaluation (JEE) and Performance Veterinary Services (PVS) - Financing - Commitment to sharing of genetic & biological data & specimens |
| Risk environment | Overall risk environment and country vulnerability to biological threats   - Political and security risk - Socio-economic resilience - Infrastructure adequacy - Environmental risks - Public health vulnerabilities |
